# Prediction of hepatic metastasis in esophageal cancer based on machine learning

**DOI:** 10.1038/s41598-024-63213-6

**Published:** 2024-06-24

**Authors:** Jun Wan, Yukai Zeng

**Affiliations:** 1 Department of Emergency surgery, Yangtze University Jingzhou Hospital, jingzhou, China; 2 Department of Thoracic Surgery, China-Japan Union Hospital of Jilin University, No. 126 Xiantai street, Changchun, Jilin China

**Keywords:** Hepatic metastasis, Esophageal cancer, Machine learning, Online calculator, Oesophageal cancer, Cancer, Cancer models

## Abstract

This study aimed to establish a machine learning (ML) model for predicting hepatic metastasis in esophageal cancer. We retrospectively analyzed patients with esophageal cancer recorded in the Surveillance, Epidemiology, and End Results (SEER) database from 2010 to 2020. We identified 11 indicators associated with the risk of liver metastasis through univariate and multivariate logistic regression. Subsequently, these indicators were incorporated into six ML classifiers to build corresponding predictive models. The performance of these models was evaluated using the area under the receiver operating characteristic curve (AUC), accuracy, sensitivity, and specificity. A total of 17,800 patients diagnosed with esophageal cancer were included in this study. Age, primary site, histology, tumor grade, T stage, N stage, surgical intervention, radiotherapy, chemotherapy, bone metastasis, and lung metastasis were independent risk factors for hepatic metastasis in esophageal cancer patients. Among the six models developed, the ML model constructed using the GBM algorithm exhibited the highest performance during internal validation of the dataset, with AUC, accuracy, sensitivity, and specificity of 0.885, 0.868, 0.667, and 0.888, respectively. Based on the GBM algorithm, we developed an accessible web-based prediction tool (accessible at https://project2-dngisws9d7xkygjcvnue8u.streamlit.app/) for predicting the risk of hepatic metastasis in esophageal cancer.

## Introduction

Esophageal cancer (EC), which occupies the ninth position in terms of global cancer prevalence, is the sixth most common cause of cancer mortality^1^. Annually, it is responsible for the demise of over half a million individuals worldwide^2,3^. From a histological viewpoint, the disease mainly bifurcates into esophageal squamous cell carcinoma (ESCC) and esophageal adenocarcinoma (EAC), each exhibiting unique patterns of metastasis that typically manifest at different stages of the disease progression^4^. Due to the predominantly asymptomatic nature of the early stages, esophageal cancer diagnosis often occurs at an advanced phase, where it is commonly accompanied by distant metastatic spread^5^. Patterns of metastasis in esophageal cancer can be classified into three major types: lymphatic, hematogenous, and direct diffusion. The latter typically becomes evident in the advanced stages, marked by tumor invasion into adjacent structures following penetration through the esophageal adventitia. Hematogenous metastasis is primarily secondary to lymph node involvement, facilitating the tumor’s spread to distant organs via the vascular system^6^. The lymphatic pathway, however, is recognized as the principal vector for metastatic dissemination in esophageal cancer, critically affecting patient prognosis and contributing to pertinent prognostic considerations^7,8^.

Metastatic sites of esophageal cancer encompass the liver, brain, lungs, bones, and others. However, liver metastasis in esophageal cancer engenders a substantial impact on patient prognosis. Not only does it signal advanced-stage disease, but it also portends a poor prognosis, resulting in metabolic disorders due to liver dysfunction, circulatory problems stemming from liver failure, pain, weight loss, and the potential development of multiple organ dysfunction syndrome (MODS) in the advanced stages^25^.

In light of this, advanced machine learning (ML) models were employed in this study. In comparison to traditional logistic models, machine learning techniques unlock richer information within extensive datasets, thus achieving superior outcome prediction accuracy^10^. ML technology has already found wide-ranging applications in science and society, ranging from driverless cars to board games to decision-making processes^11^. In the field of biomedicine, the emergence of big data in healthcare^12,13^ presents tremendous potential for ML to comprehend disease and health. Consequently, ML has been integrated into clinical diagnostics, precision therapeutics, and health monitoring^14^.

Given that patients with esophageal cancer exhibit varying clinical-pathological stages and receive different treatments, prognostic outcomes also differ significantly. Unfortunately, limited research currently focuses on hepatic metastasis metastasis in advanced esophageal cancer, thereby posing challenges for clinical decision-making among physicians^24^. Therefore, the objective of this research is to formulate and validate a machine learning model characterized by its strong predictive capabilities, and to integrate this model into an accessible web-based tool designed to facilitate the prediction of liver metastasis risk in individuals diagnosed with esophageal cancer.

## Materials and methods

### Study population

**Figure 1:**
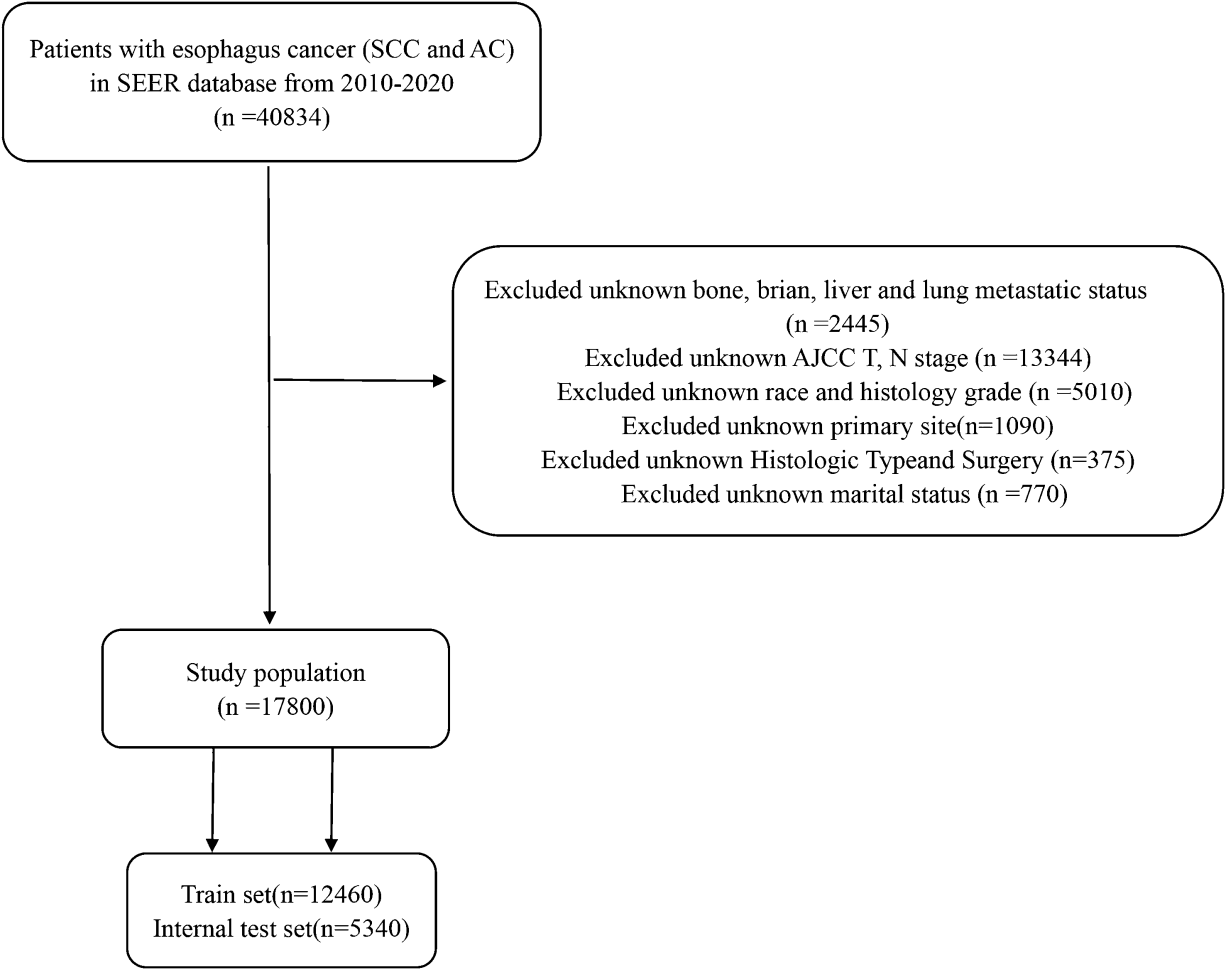
The study flow chart of case screening.

In the study, we used SEER*stat 8.4.1 software to download the patients’ data from the SEER database. Patients diagnosed with esophageal cancer (SCC and AC) between 2010 and 2020 were involved in this study. Exclusion criteria were detailed as follows: (1) Excluded unknown bone, brian, liver and lung metastatic status; (2) Excluded unknown AJCC T, N stage; (3) Excluded unknown race and histology grade; (4) Excluded unknown primary site; (5) Excluded unknown Histologic Type and Surgery; (6) Excluded unknown marital status. A study flow chart of case screening was presented in Fig. 1.

### Data selection

In this study, 16 variables related to the clinicopathology and demographics of patients were selected for analysis. Demographic variables included age, sex, marital status, race. Clinicopathological variables included primary site, tumor histology, tumor grade, T stage, N stage, surgery, radiation, chemotherapy, brain metastasis, bone metastasis, lung metastasis, liver metastasis. According to the ICD-O-3 codes, histological types of esophageal cancere divided into 2 categories, including adenocarcinoma (8140–8573), squamous cell carcinoma (8050–8082). All esophageal cancer patients were staged according the AJCC 8th edition guidelines and SEER staging information. In addition, X-tile software was used to calculate cut-off value of age.

### Data pre-processing and feature engineering

All statistical analyses were conducted with Python3.8, SPSS 23. In this study We performed a logistic regression analysis on data collected in the SEER database to identify suitable variables for machine learning model by using SPSS 23 software. Significant variables from HM patients were identified by univariate logistic regression analysis (P < 0.05). Then, these variables were enclosed within multivariate logistic regression analysis, and variables with a P < 0.05 in multivariate logistic regression analysis were subjected for further analysis of ML model. Correlation analysis was used to analyze the correlation among the selected features. Since this data set is an unbalanced data set, the over-sampling method were adopted for data processing^15^. The key of this method is to oversampling the data samples of small classes to increase the number of data samples of small classes to improve the accuracy of the model. Meanwhile, to compare the importance of each feature, we extract the feature importance of each variable in the machine learning model according to the Permutation Importance principle^16,17^.

### Model establishment and evaluation

Data from the SEER database were randomly assigned to train set and internal test set in a ratio of 3:7. Six commonly used classifier algorithms were chosen to this study, including three ensemble algorithms^11^ Random Forest (RF), Gradient Boosting ine (GBM), eXtreme gradient boosting (XGB) and three simple classification algorithms Logistic Regression (LR), Decision tree (DT), Naive Bayes classifiers (NBC). The ML models were trained using Python software. In the training group, all SEER data was divided into 10 parts for 10 × cross-validation^20^. For the internal test group data is directly imported into the built model for verification. The area under the receiver operating characteristic curve (AUC), sensitivity, specificity, accuracy and F-score were evaluated indicators of ML algorithms. The probability density plot and clinical utility curve (CUC) was utilized to examine clinical applicability. Furthermore, based on the best-performing model, we built a web-based online calculator.

### Ethical disclosure statement

The authors stated that no human or animal experiments were adopted in this study.

## Results

### Clinical characteristics of patients

In evaluating the train (N = 12,460) and test (N = 5340) sets of esophageal cancer patients, no significant differences were observed in terms of age distribution, sex, marital status, race, tumor characteristics, and treatments received, with P-values exceeding 0.05 for all compared variables. The most common tumor location was the lower third of the esophagus, and adenocarcinoma was the prevalent histology type. The rates of the various interventions and metastases, including liver metastasis (9.3% in training vs. 9.0% in testing), were similarly distributed between the two sets, indicating a well-matched cohort for further predictive analysis (Table 1).

**Table 1:** Clinical and pathological characteristics of train set and internal test set.

Variables	Training set	Internal test	Value of P
	(N = 12,460)	(N = 5340)	
Age			
< 70	7456 (59.8%)	3092 (57.9%)	0.054
70–80	3393 (27.2%)	1529 (28.6%)	
≥ 80	1611 (12.9)	719 (13.5%)	
Sex			
Female	2595 (20.8%)	1101 (20.6%)	0.753
Male	9865 (79.2%)	4239 (79.4%)	
Marital status			
Unmarried	2171 (17.4%)	953 (17.8%)	0.497
Married	10,289 (82.6%)	4387 (82.2%)	
Race			
Asian or Pacific Islander	640 (5.1%)	286 (5.4%)	0.705
White	10,740 (86.2%)	4585 (85.9%)	
Black	999 (8.0%)	427 (8.0%)	
American Indian/Alaska Native	81 (0.7%)	42 (0.8%)	
Primary site			
Upper third of esophagus	922 (7.4%)	400 (7.5%)	0.532
Middle third of esophagus	2324 (18.7%)	953 (17.8%)	
Lower third of esophagus	8668 (69.6%)	3737 (70.0%)	
Overlapping lesion of esophagus	546 (4.4%)	250 (4.7%)	
Tumor histology			
Adenocarcinoma	8628 (69.2%)	3645 (68.3%)	0.192
Squamous–cell carcinoma	3832 (30.8%)	1695 (31.7%)	
Tumor grade			
Grade I	791 (6.3%)	335 (6.3%)	0.912
Grade II	5546 (44.5%)	2395 (44.9%)	
Grade III	6123 (49.1%)	2610 (48.9%)	
T stage			
T1	2648 (21.3%)	1144 (21.4%)	0.664
T2	1829 (14.7%)	819 (15.3%)	
T3	6325 (50.8%)	2681 (50.2%)	
T4	1658 (13.3%)	696 (13.0%)	
N stage			
N0	4887 (39.2%)	2165 (40.5%)	0.416
N1	5748 (46.1%)	2419 (45.3%)	
N2	1352 (10.9%)	562 (10.5%)	
N3	473 (3.8%)	194 (3.6%)	
Surgery			
No	7849 (63.0%)	3401 (63.7%)	0.378
Yes	4611 (37.0%)	1939 (36.3%)	
Radiation			
No	3875 (31.1%)	1643 (30.8%)	0.661
Yes	8585 (68.9%)	3697 (69.2%)	
Chemotherapy			
No	3192 (25.6%)	1390 (26.0%)	0.565
Yes	9268 (74.4%)	3950 (74.0%)	
Brain metastasis			
No	12,347 (99.1%)	5285 (99.0%)	0.436
Yes	113 (0.9%)	55 (1.0%)	
Bone metastasis			
No	646 (5.2%)	285 (5.3%)	0.675
Yes	11,814 (94.8%)	5055 (94.7%)	
Lung metastasis			
No	11,739 (94.2%)	5055 (94.7%)	0.234
Yes	721 (5.8%)	285 (5.3%)	
Liver metastasis			
No	11,300 (90.7%)	4857 (91.0%)	0.576
Yes	1160 (9.3%)	483 (9.0%)	

### Univariable and multivariable logistic regression analysis

11 risk factors associated with hepatic metastasis including age, primary site, tumor histology, tumor grade, T stage, N stage, surgery, radiation, chemotherapy, bone metastasis, lung metastasis were identified using univariable and multivariable LR analysis (P < 0.05, Table 2). Based on these risk factors, we developed six different models using machine learning (ML) algorithms in this study.

**Table 2:** Univariate analysis and multivariate logistic regression analysis of variables.

Variables	Univariate logistic analysis		Multivariate logistic analysis	
	OR (95% CI)	P-value	OR (95% CI)	P-value
Age				
< 70	Reference		Reference	
70–80	0.724 (0.626–0.838)	< 0.001	0.694 (0.585–0.822)	< 0.001
≥ 80	0.678 (0.555–0.830)	< 0.001	0.574 (0.455–0.725)	< 0.001
Sex				
Female	Reference		Reference	
Male	1.703 (1.434–2.024)	< 0.001	1.192 (0.975–1.456)	0.086
Marital status				
Unmarried	Reference		Reference	
Married	0.854 (0.733–0.995)	0.043	1.066 (0.888–1.208)	0.495
Race				
Asian or Pacific Islander	Reference		Reference	
White	1.429 (1.045–1.954)	0.025	0.992 (0.687–1.433)	0.966
Black	1.211 (0.828–1.772)	0.324	1.056 (0.683–1.633)	0.807
American Indian/Alaska Native	1.693 (0.794–3.612)	0.173	0.987 (0.399–2.440)	0.977
Primary site				
Upper third of esophagus	Reference		Reference	
Middle third of esophagus	1.892 (1.248–2.867)	0.003	1.683 (1.078–2.627)	0.022
Lower third of esophagus	3.837 (2.618–5.624)	< 0.001	3.015 (1.978–4.708)	< 0.001
Overlapping lesion of esophagus	4.850 (3.091–7.610)	< 0.001	2.253 (1.359–3.735)	0.002
Tumor histology				
Adenocarcinoma	Reference		Reference	
Squamous–cell carcinoma	0.404 (0.343–0.474)	< 0.001	0.438 (0.350–0.549)	< 0.001
Tumor grade				
Grade I	Reference		Reference	
Grade II	2.264 (1.543–3.322)	< 0.001	1.808 (1.175–2.783)	0.007
Grade III	3.343 (2.288–4.884)	< 0.001	1.722 (1.123–2.641)	0.013
T stage				
T1	Reference		Reference	
T2	0.485 (0.379–0.621)	< 0.001	0.538 (0.406–0.712)	< 0.001
T3	0.704 (0.600–0.827)	< 0.001	0.622 (0.510–0.759)	< 0.001
T4	2.606 (2.191–3.099)	< 0.001	1.178 (0.953–1.456)	0.129
N stage				
N0	Reference		Reference	
N1	2.494 (2.153–2.888)	< 0.001	1.787 (1.501–2.127)	< 0.001
N2	1.450 (1.145–1.836)	0.002	1.450 (1.100–1.911)	0.008
N3	3.579 (2.730–4.691)	< 0.001	1.987 (1.420–2.779)	< 0.001
Surgery				
No	Reference		Reference	
Yes	0.031 (0.021–0.046)	< 0.001	0.037 (0.024–0.056)	< 0.001
Radiation				
No	Reference		Reference	
Yes	0.243 (0.214–0.275)	< 0.001	0.269 (0.231–0.314)	< 0.001
Chemotherapy				
No	Reference		Reference	
Yes	0.792 (0.694–0.904)	0.001	1.297 (1.092–1.541)	0.003
Brain metastasis				
No	Reference		Reference	
Yes	4.476 (2.990–6.700)	< 0.001	1.525 (0.962–2.417)	0.073
Bone metastasis				
No	Reference		Reference	
Yes	7.165 (6.028–8.518)	< 0.001	2.427 (1.984–2.968)	< 0.001
Lung metastasis				
No	Reference		Reference	
Yes	10.717 (9.108–12.611)	< 0.001	4.600 (3.805–5.562)	< 0.001

### Correlation analysis and Importance of features on prediction

**Figure 2:**
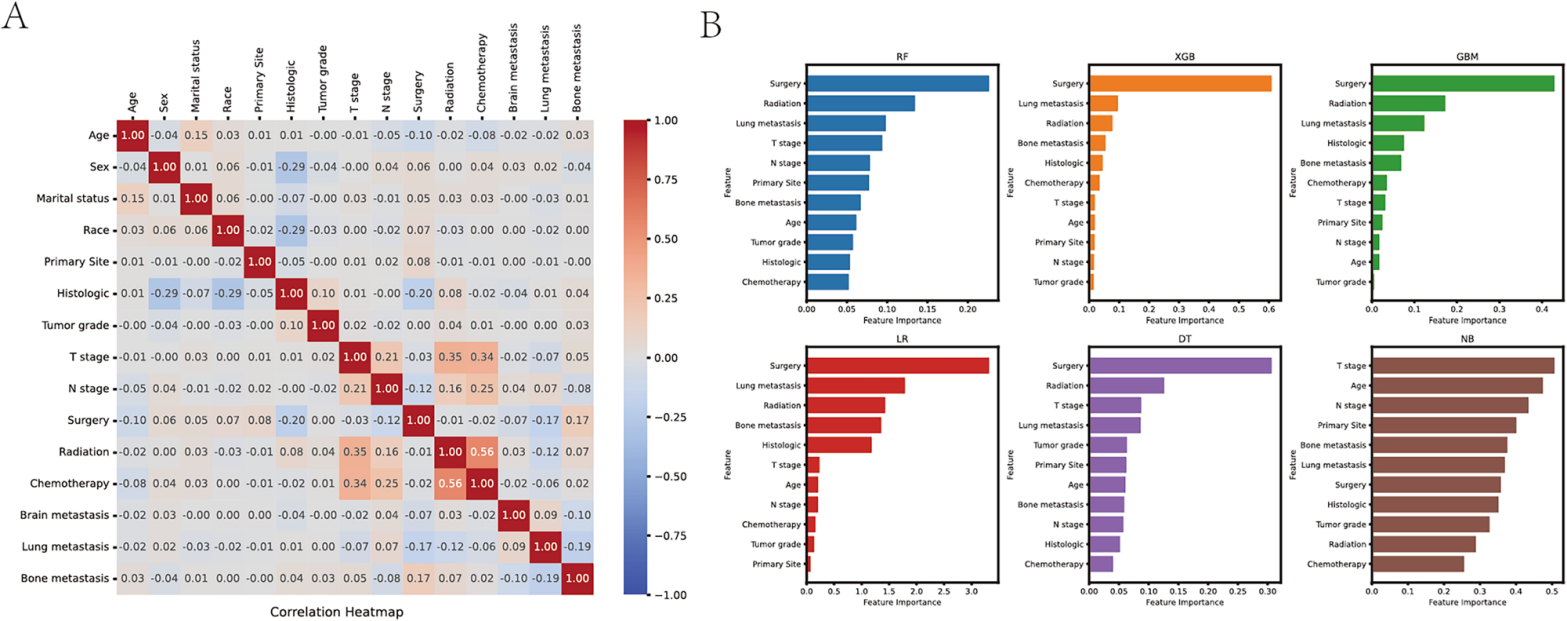
(**A**) Heat map of the correlation of features. (**B**) Feature importance of different models.

In order to assess the level of correlation between factors, correlation analysis is commonly employed. In this study, we utilized Spearman correlation analysis to examine the independence between data features. A correlation heat map was generated, as depicted in Fig. 2A, which depicted the absence of significant correlation among the 15 features under investigation. Figure 2B presents the significance of features extracted from each machine learning algorithm. The variables identified through univariate and multivariate logistic analysis have all played a remarkable role in predicting outcomes across the six models. Notably, surgery consistently emerged as the most influential feature in the majority of prediction models, underscoring its significant impact on hepatic metastasis in esophageal cancer. In most algorithms, T stage, age, primary, N stage and tumor grade ranked the last five, with no significant difference in their contributions to the model. Lung metastasis, radiation, bone metastasis, histology, chemotherapy, T stage, age, primary, N stage and tumor grade are arranged in descending order in GBM model.

### Model performance

**Figure 3:**
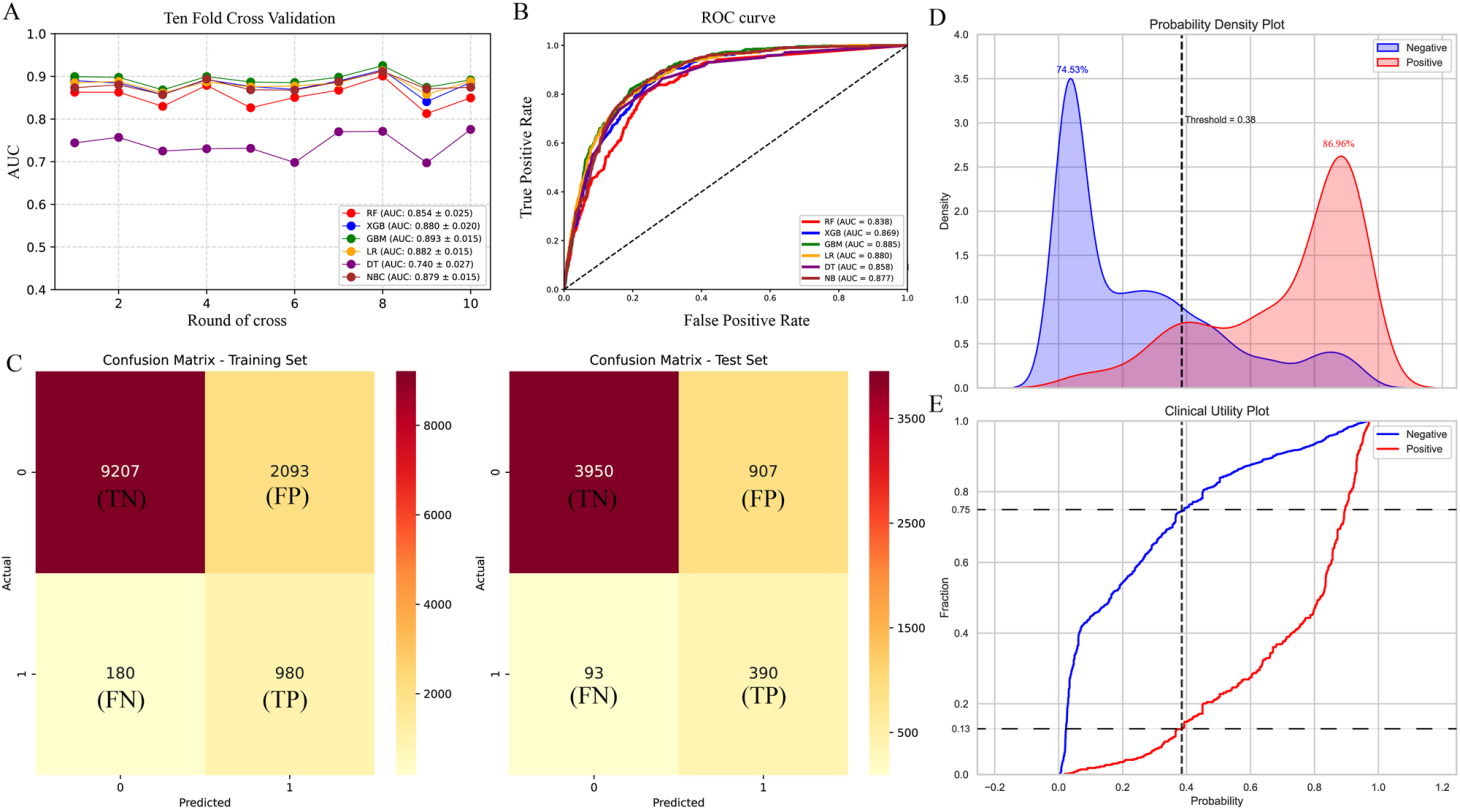
(**A**) Ten-fold cross-validation results of different machine learning models. (**B**) The roc curves of different machine learning models in internal test set. (**C**) The confusion matrix of the GBM model in the train set and the internal test set. *TP* true positive, *TN* true negative, *FP* false positive, *FN* false negative. (**D**) Probability density plot of gradient boosting machine model. (**E**) The clinical impact curve of gradient boosting machine model.

The performance of the six predictive models is described in Fig. 3A,B and Table 3. Internal ten-fold cross-validation (Fig. 3A) showed that GBM model performed best among the six models with an average AUC of 0.893, followed by the LR model (AUC = 0.882). Internal test validation was shown in Table 3 and Fig. 3B. Interestingly, the GBM model also achieves the best AUC score (0.885) in the internal test validation and the score of accuracy, sensitivity (recall rate) and specificity were 0.868, 0.667 and 0.888, respectively. The confusion matrix (Fig. 3C) of the GBM model in the training set and the test set indicated its high accuracy. The probability density plot (Fig. 3D) depicting predictive distribution showed that the AUC was highest when the predictive score was 0.38. The CUC plot (Fig. 3E) also showed good clinical applicability.

**Table 3:** Prediction performance of different models.

	RF	XGB	GBM	LR	DT	NB
AUC	0.83839	0.869439	0.88486	0.880388	0.858161	0.876666
Accuracy	0.864981	0.873783	0.867978	0.797566	0.827903	0.801498
Sensitivity	0.467909	0.565217	0.666667	0.805383	0.73913	0.807453
Specificity	0.904468	0.904468	0.887997	0.796788	0.83673	0.800906
F-score	0.385337	0.447541	0.477391	0.418505	0.437232	0.423913

### Web predictor

**Figure 4:**
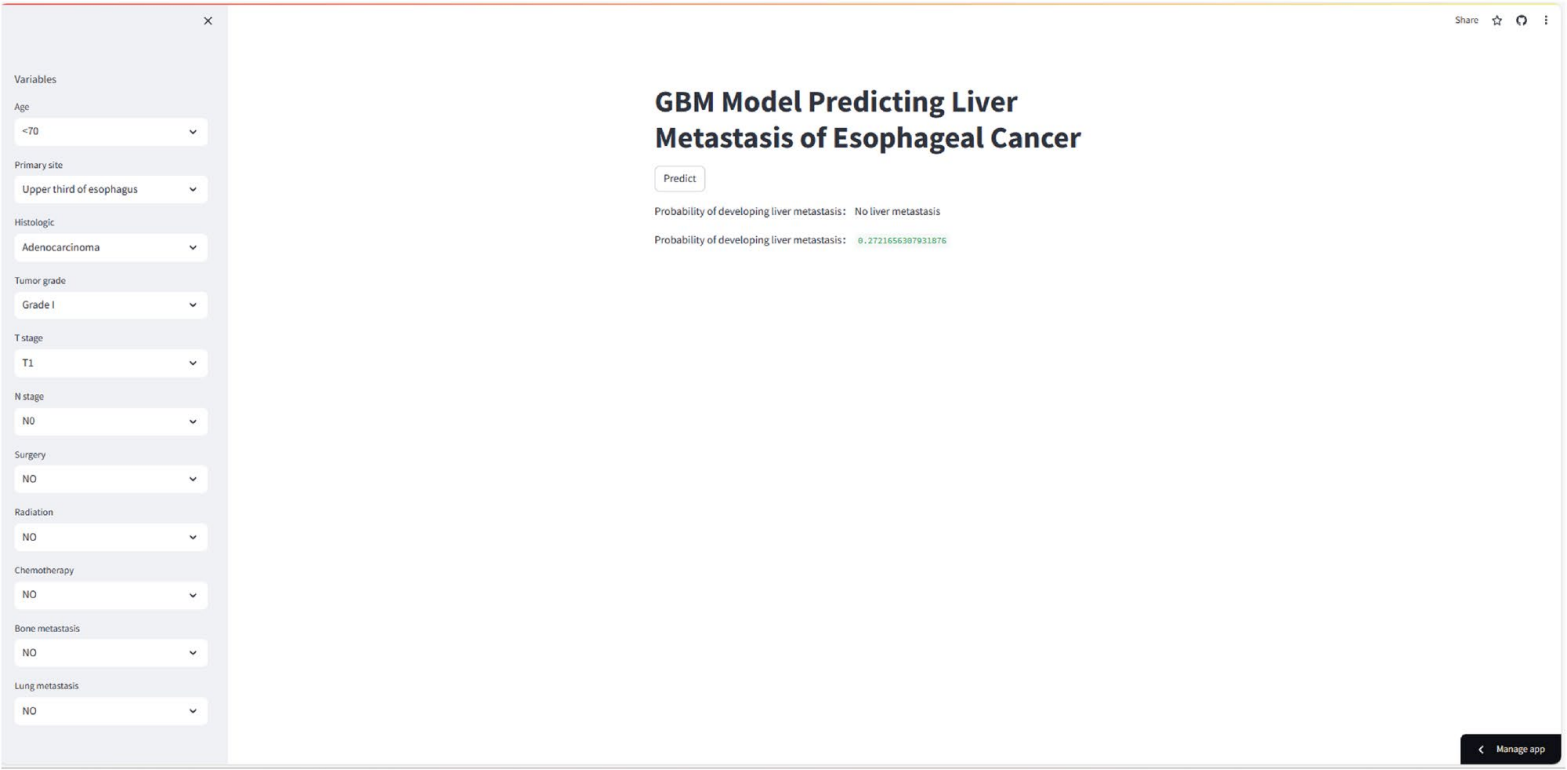
A web predictor for predicting HM in EC.

This study aimed to develop a web predictor utilizing the GBM model, which exhibited superior predictive performance for hepatic metastasis in patients with esophageal cancer. The primary objective of this web predictor is to provide doctors with a valuable tool for making more precise clinical decisions. By inputting the relevant variables associated with hepatic metastasis into the web predictor, healthcare professionals can conveniently calculate the odds of hepatic metastasis in patients with esophageal cancer. For easy access, the web predictor can be accessed at the following link: (https://project2-dngisws9d7xkygjcvnue8u.streamlit.app/). Please refer to Fig. 4 for further details.

## Discussion

Esophageal cancer is a remarkably fatal malignancy, with a prevalence of distant metastases reaching up to 42% in newly diagnosed patients, prominently affecting the liver as the most frequently involved organ^26–28^. The effective treatment and comprehensive management of metastatic esophageal cancer necessitate a multimodal strategy, which continues to pose significant challenges. Therefore, it is of crucial significance for clinical decision-making to identify high-risk factors of esophageal cancer and accurately predict whether patients will develop liver metastasis based on their individual and unique clinical and pathological characteristics.

Currently, the HM of advanced esophageal cancer remains understudied in the scientific literature. Prognostic research in this domain is predominantly focused on two key aspects. Firstly, there is a conspicuous paucity of exploratory investigations into the high-risk prognostic factors associated with esophageal cancer. Additionally, further exploration of the interrelationships among these independent prognostic factors is noticeably lacking. Secondly, there is a dearth of research on HM models for advanced esophageal cancer that leverage the immense potential of big data. Consequently, there is an urgent need for comprehensive studies in these areas to contribute to an improved understanding and accurate prognostication of advanced esophageal cancer.

Some studies believe that smoking and drinking are the most common risk factors for male esophageal cancer^29^. Some previous studies^30^ have also shown that for cancer patients, the degree of tissue differentiation, pathological N-stage, vascular invasion, and neuroinvasion are recognized factors that affect the prognosis of patients with esophageal cancer^31–34^. The conclusions of these studies lacked the support of big data and did not address the prediction on HM of advanced esophageal cancer. Based on big data analysis of SEER database, our study screened out independent high risk factors associated with HM by logistic regression analysis. This study included 15 clinically common relevant factors associated with advanced esophageal cancer with liver metastasis, which are: age, sex, Marital status, Race, Primary Site, Tumor histology, Tumor grade, T stage, N stage, Surgery, Radiation, Chemotherapy, Brain metastasis, Bone metastasis, Lung metastasis. To identify the independence between features, we obtained a correlation heat map by Spearman correlation analysis. There was no strong correlation among these 15 features by the Fig. 2A. Moreover, 11 independent high risk factors related to liver metastasis were screened by logistic regression analysis, which were as follows: age, Primary Site, Tumor histology, Tumor grade, T stage, N stage, Surgery, Radiation, Chemotherapy, Bone metastasis, Lung metastasis.

Undoubtedly, the construction of prediction models for HM of advanced esophageal cancer is equally significant to the exploration of independent high risk factors in this context. Presently, there is a notable dearth of studies focused on risk factors in esophageal cancer patients with distant organ metastases^35^. For instance, Tang et al. previously constructed a nomogram to predict the survival of patients with metastatic esophageal cancer; however, this study encompassed metastases to all anatomical sites, without specifically exploring a prediction model for predicting the risk of distant metastasis^36^. Similarly, Cheng et al. established models for predicting both the risk and survival of esophageal cancer patients, albeit those specifically tailored to brain metastasis^37^. Furthermore, Guo et al. provided detailed characteristics and explored risk and prognostic factors for patients with liver metastasis, yet they did not develop any predictive tools^38^. Considering that liver metastasis represents the most common site of distant spread, conducting a comprehensive investigation specifically targeting esophageal cancer patients with liver metastasis assumes paramount clinical importance.

Previous studies have constructed nomograms to predict EC metastasis based on traditional logistic models. However, the limitations of this method in prediction accuracy and processing big data have made it difficult to make great breakthroughs in precision medicine^9,10^. And traditional research cannot exploration the interaction between different independent high risk factors^18,19^. In contrast, our study can better document complex associations between different independent high risk factors, thereby improving the accuracy of the model^20^. Previous studies have used nomogram methods to build a model for predicting the metastasis of patients with esophageal cancer based on the data of patients with esophageal cancer in the SEER database, but these studies did not involve the establishment of a predicting model for HM of advanced metastatic esophageal cancer by ML^21^.

We then constructed six prediction models using ML, Internal ten-fold cross-validation (Fig. 3A) showed that GBM model performed best among the six models. Leveraging these findings, we have successfully devised an openly accessible online calculator (https://project2-dngisws9d7xkygjcvnue8u.streamlit.app/) based on the GBM model. The model we have developed accurately predicts patients' risk of HM based on various clinical indicators. Clinicians can access this model through the provided website to input patient information and obtain corresponding predictions of hepatic metastases, thereby facilitating clinical decision-making.

Our research has the following advantages. Firstly, this study established a statistical model based on machine learning that can predict the HM of patients with EC. To the best of our knowledge, we are the first to use ML to construct a prediction model of LM of EC. This model is more reliable than the traditional nomogram prediction model. And this work expanded our knowledge of advanced EC. Second, our study further explores the relationship between different independent high risk factors, which provides a new direction for future clinical research. In other words, clinical research should not only explore the metastasis of patients, but also explore the correlation between different independent high risk factors, so as to better find the relationship between these factors and further eliminate the factors that are not conducive to the metastasis of patients during perioperative period.

Meanwhile, this study has some limitations. First, Current machine learning is almost entirely statistical or black-box, bring severe theoretical limitations to its performance^23^. Second, this study is a single-center study with limited number of patients included, and the application of machine learning model on large data sets can obtain more stable results^22^. Therefore, in subsequent studies, multi-center data can be added for training and external verification, so as to obtain a more reliable prediction model. Third, this study did not include neoadjuvant therapy, surgical methods, circulating tumor DNA and other factors that may affect the long-term prognosis of patients with esophageal cancer. In the future, with the continuous improvement of the database, we will incorporate more correlation parameters associated with the HM of EC into the web predictor to improve its adaptability.

## Conclusion

In summary, this study built a machine learning model for predicting liver metastasis of esophageal cancer based on 11 clinicopathological features commonly seen in clinical work, among which GBM model performed best. GBM model can be used to predict liver metastasis of esophageal cancer, and then help clinicians to make more accurate treatment plan for patients with esophageal cancer.

## Data Availability

The datasets used and analyzed during the current study are available from the corresponding author on reasonable request.
